# The anticancer activity of lytic peptides is inhibited by heparan sulfate on the surface of the tumor cells

**DOI:** 10.1186/1471-2407-9-183

**Published:** 2009-06-15

**Authors:** Bodil Fadnes, Øystein Rekdal, Lars Uhlin-Hansen

**Affiliations:** 1 Department of Medical Biochemistry, Institute of Medical Biology, University of Tromsø, Norway; 2 Department of Anatomy and Pathology, University Hospital of North Norway, Tromsø, Norway; 3 Department of Pathology, Institute of Medical Biology, University of Tromsø, Tromsø, Norway; 4 Lytix Biopharma, Tromsø Science Park, N-9294, Tromsø, Norway

## Abstract

**Background:**

Cationic antimicrobial peptides (CAPs) with antitumor activity constitute a promising group of novel anticancer agents. These peptides induce lysis of cancer cells through interactions with the plasma membrane. It is not known which cancer cell membrane components influence their susceptibility to CAPs. We have previously shown that CAPs interact with the two glycosaminoglycans (GAGs), heparan sulfate (HS) and chondroitin sulfate (CS), which are present on the surface of most cells. The purpose of this study was to investigate the role of the two GAGs in the cytotoxic activity of CAPs.

**Methods:**

Various cell lines, expressing different levels of cell surface GAGs, were exposed to bovine lactoferricin (LfcinB) and the designer peptide, KW5. The cytotoxic effect of the peptides was investigated by use of the colorimetric MTT viability assay. The cytotoxic effect on wild type CHO cells, expressing normal amounts of GAGs on the cell surface, and the mutant pgsA-745, that has no expression of GAGs on the cell surface, was also investigated.

**Results:**

We show that cells not expressing HS were more susceptible to CAPs than cells expressing HS at the cell surface. Further, exogenously added heparin inhibited the cytotoxic effect of the peptides. Chondroitin sulfate had no effect on the cytotoxic activity of KW5 and only minor effects on LfcinB cytotoxicity.

**Conclusion:**

Our results show for the first time that negatively charged molecules at the surface of cancer cells inhibit the cytotoxic activity of CAPs. Our results indicate that HS at the surface of cancer cells sequesters CAPs away from the phospholipid bilayer and thereby impede their ability to induce cytolysis.

## Background

Cationic antimicrobial peptides (CAPs), also termed host defense peptides, play a part in the innate immune system [1]. CAPs can be assigned into two broad groups based on their target specificity, one group comprising antimicrobial activity, such as defensins [2,3], and cecropins [4], which have specificity for prokaryotic cell membranes, and a second group, the venom peptides with activity against both prokaryotic and eukaryotic cell membranes e.g. melittin [5] and mastoparan [6]. In addition, some of the CAPs show selective activity against cancer cells. These peptides act via a non-receptor-mediated pathway and constitute a promising group of novel anticancer agents with a new mode of action and a broad spectrum of anticancer activity. Several studies, included our earlier reported *in vivo *studies, indicate that CAPs may have a potential for local treatment of solid tumors [7-10]. Compared to conventional chemotherapy these CAPs display a higher specificity for cancer cells versus normal cells [11,12]. In addition, the peptides are able to kill cancer cells that have become resistant to conventional chemotherapeutics [13-16]. It has also been reported that certain CAPs have the potential to enhance the cytotoxic activity of different chemotherapeutics against multi-drug resistant tumor cells [13-15]. The reason why CAPs display activity against chemoresistant cancer cells probably lies in their rapid mode of action against the plasma membrane, resulting in lysis of the cells [17]. Most of the CAPs selective for cancer cells interact with the target membrane through the "carpet model", where the peptides align parallel to the outer membrane surface and permeate the membrane after a threshold concentration of peptides have been reached [18,19]. In addition, some CAPs can also trigger apoptosis in cancer cells via mitochondrial membrane disruption [20].

It is not yet clarified which components in the plasma membrane render cancer cells more susceptible to CAPs than non-malignant cells. Due to the cationic nature of the peptides, the interaction with the cell surface of the target cells is most likely facilitated by negatively charged molecules in the plasma membrane. Whereas the plasma membranes of non-malignant eukaryotic cells consist primarily of zwitterionic and neutral phospholipids [21], a number of studies have revealed that the outer membrane leaflet of cancer cells is more negatively charged than their normal counterparts. An elevated expression of the anionic phospholipid phosphatidylserine in the outer leaflet of the plasma membrane has been found in several types of cancer cell lines [22-25]. Alterations in the carbohydrate portion of glycoproteins and glycolipids, including increased sialylation, also contributes to a more negatively charged tumor cell surface [26]. The enhanced expression of terminal sialic acids on cell surface N-linked glycans and O-linked glycans has been reported as a characteristic in a variety of cancers [27].

Proteoglycans (PGs) are also expressed on the cell surface. These are characterized by highly negatively charged glycosaminoglycan (GAG) side chains attached to a core protein [28]. Two major classes of GAG side chains are heparan sulfate (HS) and chondroitin sulfate (CS). These molecules consist of a linear repeat of up to 100 disaccharide units [29,30]. Both HS and CS are highly sulfated with an average number of approximately 1.2 and 1.0 negatively charged sulfate groups per disaccharide unit, respectively [31,32]. The sulfate groups make therefore the PGs some of the most anionic molecules on the cell surface. It has been shown that several cancer cells have a different expression of cell surface PGs, compared to their normal counterpart cells [33-35]. In addition, it has been shown that the degree and pattern of sulfation of the GAG chains may be altered during malignant transformation [36,37].

One member of the CAP family is bovine lactoferricin (LfcinB). This peptide displays cytotoxic activity against a variety of cancer cells *in vitro*, without harming normal cells [8,38]. In addition, LfcinB is able to prevent tumor growth and metastasis in several mouse models [7,8,39]). Previous studies have revealed that LfcinB induces cell death in tumor cells by targeting the plasma membrane and after internalization, the mitochondria [8].

We have previously shown that LfcinB binds to GAGs [40]. Since the fundamental activity of antimicrobial peptides stems from their ability to interact with negatively charged membrane molecules, we hypothesized that GAGs at the surface of target cells may interact with CAPs and enhance their cytotoxic activity. In the present study the role of GAGs in the cytotoxic activity of LfcinB was thus investigated. LfcinB is a 25-mer peptide with a disulfide bridge that forms a stabilized amphipatic β-sheet structure. In addition, the KW5 peptide, a 21-mer peptide designed to adopt an idealized amphipathic α-helix was also included in the study.

## Methods

### Reagents

The LfcinB peptide was provided by the Centre for Food Technology (Queensland, Australia). All Fmoc-amino acids, Fmoc-resins and chemicals used during peptide synthesis, cleavage and precipitation were purchased from PerSeptive (Hertford, UK), Fluka (Buchs, Switzerland) and Sigma-Aldrich (St. Louis, MO). Fetal bovine serum (FBS) was obtained from Biochrom KG (Berlin, Germany), and L-glutamine from Gibco (Paisley, Scotland). MTT (3-(4, 5-dimethylthiazol-2-yl)-2.5-diphenyl tetrazolium bromide) was obtained from Sigma-Aldrich (Oslo, Norway). Heparitinase (EC 4.2.2.8) and chondroitinase ABC (EC 4.2.2.4) were from Seikagaku Corporation (Chuo-ku, Tokyo, Japan). Heparin (H-3393) and chondroitin sulfate (C-4384) were obtained from Sigma-Aldrich (Oslo, Norway). [^35^S]Sulfate (code SJS-1) was purchased from Amersham Biosciences (Buckinghamshire, England).

The lymphoma cell lines KMS-5, KMM-1 and Sudhl-4 were a kind gift from Mark Raffeld, Hematophathology Section, Laboratory of Pathology, National Cancer Institute, National Institutes of Health, Bethesda, MD. The lymphoma cell lines Raji and Ramos were provided by Dr. Michael Norcross, Division of Hematologic Products, Center for Biologics Evaluation and Research, Food and Drug Administration, Bethesda, MD, while the lymphoma cell line U-266, colon carcinoma cell line HT-29 and the melanoma cell line FEMX were purchased from ATCC. Jeffery D. Esko, Department of Cellular and Molecular Medicine, University of California, San Diego, USA, kindly provided us with the mutant Chinese hamster ovary cell line pgsA-745, which does not express GAGs at the cell surface, as well as the wild type CHO-K1 that expresses normal amounts of GAGs [41,42].

### Peptide synthesis, purification and analysis

The peptide KW5, ((KAAKKAA)_3 _W 7,9,16), was synthesized by solid-phase methods using standard Fmoc chemistry on a Pioneer Peptide synthesizer (Applied Biosystems, Foster City, CA). Crude peptides were purified by preparative RP-HPLC (Waters, Milford, MA) using a C_18 _column (Delta-Pak™ C18, 100 Å, 15 μm, 25–100 mm), and analyzed on an analytical C_18 _HPLC column (Delta-Pak™ C18, 100 Å, 5 μm, 3.9 × 150 mm) (Waters, Milford, MA). The purity of the peptides was found to be > 95%. Peptide characterization was done by positive ion electrospray ionization mass spectrometry on a VG quattro quadrupole mass spectrometer (VG Instruments Inc., Altringham, UK).

### Cell cultures

The HT-29 and FEMX cell lines were maintained as monolayer cultures in RPMI-1640 supplemented with 10% (v/v) FBS and 1% L-glutamine, while the CHO-K1 and pgsA-745 cell lines were cultured in HAM's-F12 supplemented with 10% (v/v) FBS and 1% L-glutamine. All the lymphoma cell lines were grown in suspension in RPMI-1640 medium supplemented with 10% (v/v) FBS and 1% L-glutamine. All cells were grown in tissue culture flasks in humidified atmosphere of 95% air and 5% CO_2 _at 37°C.

### Cytotoxicity assay

The colorimetric MTT viability assay was used to investigate the cytotoxic effect of the peptides. The FEMX and HT-29 cells were seeded at a concentration of 2 × 10^5 ^cell/ml, and the CHO-K1 and pgsA-745 cells at a concentration of 1.5 × 10^5 ^cell/ml in a volume of 0.1 ml in 96-well plates. The cells were allowed to adhere overnight in complete medium. Before adding different concentrations of the peptides (1–500 μg/ml) to the cells, the culture medium was removed and the cells were washed twice in serum-free culture medium. The non-adherent lymphoma cell lines were seeded at a density of 4 × 10^5 ^cells/ml using serum-free medium. The cells were incubated with the LfcinB peptide at 37°C for 24 h or with the KW5 peptide for 30 min. Due to our previous structure-activity relationship studies on lactoferrrin and LfcinB [7,43] structural parameters important for lytic activity against cancer cells are optimized in *de novo *designed peptides. Hence, the KW5 peptide is more active and kills cancer cells more efficiently than LfcinB. Cells in serum-free medium alone were used as a negative control whereas cells treated with 1% Triton X-100 in serum-free medium were used as a positive control for 100% cell death. After incubation, 10 μl (adherent cells) or 20 μl (non-adherent cells) MTT-solution (5 mg MTT per ml phosphate buffered saline) was added to each well and the incubation was continued for 2 h. A volume of 80 μl or 130 μl per well was removed from the non-adherent and the adherent cells, respectively. In order to dissolve the formazan crystals, 100 μl of 0.04 M HCl in isopropanol was added and the plates were shaken for 1 h on a Thermolyne Roto Mix (Dubuque, IA) at room temperature. The optical density was measured on a microtitre plate reader (Thermomax Molecular Devises, NJ). Cell survival was determined from the ΔA_590 _nm relative to the negative control (100% living cells) and expressed as 50% inhibitory concentration (IC_50_).

### Sodium chlorate treatment

The FEMX and HT-29 cells were seeded at a concentration of 2 × 10^5 ^cell/ml in a volume of 0.1 ml in 96-well plates. The cells were incubated for 24 h at 37°C in complete medium supplemented with 30 mM sodium chlorate, which decreases sulfation by inhibiting ATP-sulphurylase [44]. After the treatment period the culture medium containing sodium chlorate was removed and the cells were washed with serum free RMPI-1640 medium before the cytotoxic activity of the peptides were investigated using the MTT assay described above.

### Heparitinase treatment

Heparitinase purified from *Flavobacterium heparinum *was used for enzymatic cleavage of cell surface HS. Heparitinase (0.1 U) was dissolved in 100 μl 0.1% M Tris acetate (pH 7.3). FEMX and HT-29 cells were seeded at a concentration of 1 × 10^5 ^cell/ml in a volume of 0.1 ml in 96-well plates. After 48 h at 37°C the cells were treated with 0, 01 U heparitinase in serum free medium for 2 h at 37°C. Thereafter some cell cultures were washed with serum free medium before the cytotoxic activity of the peptides was investigated. In other cell cultures heparitinase was present during the whole peptide incubation period.

### Radiolabeling and isolation of ^35^S-labeled macromolecules

Cell cultures were radiolabeled for 20 h by adding [^35^S]sulfate to a final concentration of 50 μCi/ml at the time of cell plating. To be able to compare the amount of ^35^S-labeled macromolecules synthesized by the different cell lines, the cells were cultured at same level of sub-confluence. After the incubation time, the plasma membrane-associated ^35^S-labeled macromolecules were harvested by washing the cells twice with serum free-medium and subsequently incubated for 15 min at 37°C in the presence of 10 μg/ml of trypsin [45]. Free [^35^S]sulfate was removed by gel filtration on Sephadex G50 Fine columns (bed volume 4 ml, equilibrated with 0.5 M Tris/HCl, pH 8.0 and 0.15 M NaCl and eluted with dH_2_O). Aliquots from the membrane fractions were analyzed for radioactivity in a scintillation counter after the addition of Ultima Gold XR scintillation fluid. The rest of the material was immediately frozen and stored until further analysis.

### Alkali treatment and gel chromatography

The ^35^S-labeled macromolecules were subjected to alkali treatment (0.5 M NaOH over night at 45°C, followed by neutralization with 0.5 M HCl), resulting in liberation of free ^35^S-labeled GAG chains. The ^35^S-labeled macromolecules were subjected to Superose 6 gel chromatography both before and after alkali treatment. Markers for void (V_o_) and total volume (V_t_) were blue dextran and [^35^S]sulfate, respectively. The columns were run in 4 M guanidine-HCl with 0.05 M sodium acetate, pH 5.8. Fractions were collected and the radioactivity counted in a scintillation counter.

### Selective PG degradation

The ^35^S-labeled macromolecules were subjected to enzymatic treatment with chondroitinase ABC (cABC), which depolymerizes CS. Incubations with cABC were performed at 37°C overnight with 0.01 U enzyme per sample in 0.05 M Tris/HCl, 0.05 M sodium acetate, pH 8.0. The samples were analyzed on Sephadex G-50 Fine columns (bed volume 4 ml, equilibrated and eluted with the Tris/HCl buffer). In addition, parallel samples were subjected to HNO_2 _treatment at pH 1.5, in order to degrade the HS chains [46]. The samples were analyzed by Sephadex G-50 Fine columns (bed volume 4 ml, equilibrated and eluted with dH_2_O). Aliquots from the collected fractions where analyzed for radioactivity in a scintillation counter after the addition of Ultima Gold XR scintillation fluid.

## Results

### Cytotoxic effect of the peptides

**Figure 1:**
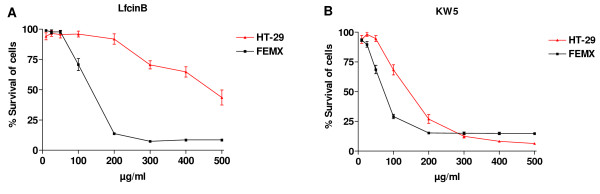
**The cytotoxic activity of LfcinB and KW5 against FEMX melanoma and HT-29 colon carcinoma cells**. The dose response curves for LfcinB (A) and KW5 (B) are plotted as percent survival of the cells against different peptide concentrations (μg/ml). The curves correspond to five experiments performed in duplicate ± SEM.

The LfcinB and KW5 peptides were tested for cytotoxic activity against the melanoma cell line FEMX and the colon carcinoma cell line HT-29. The cytotoxic activity of the peptides was determined by the colorimetric MTT viability assay. Dose-response studies revealed that the peptides displayed a significantly higher cytotoxic activity against the FEMX cells compared to the HT-29 cells (Figure 1). LfcinB showed a 3.7 fold higher activity against the FEMX cells (IC_50 _= 40 μM) compared to the HT-29 cells (IC_50 _= 148 μM). The KW5 peptide displayed a 1.8 fold higher activity against the FEMX cells (IC_50 _= 30) compared to the HT-29 cells with an IC_50 _value of 55 μM (Table 1).

**Table 1:** The cytotoxic effect of LfcinB and KW5 against FEMX and HT-29 cells.

Peptides	FEMX ^a^IC_50 _(μM)	HT-29 IC_50 _(μM)	Ratio(IC_50_)HT-29 cells/FEMX cells
LfcinB	40 ± 7	148 ± 8	3.7
KW5	30 ± 3	55 ± 14	1.8

^a^IC_50 _is the inhibitory concentration of the peptides where 50% of the cells are killed.

Data are mean ± SEM (n = 5).

### Sodium Chlorate treatment of the target cells enhances the cytotoxic effect of the peptides

**Figure 2:**
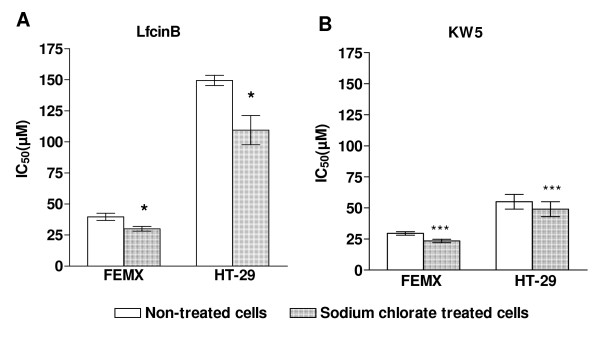
**The cytotoxic effect of LfcinB and KW5 against sodium chlorate treated and non-treated FEMX and HT-29 cells**. The results are shown as the mean IC_50 _value ± SEM of LfcinB (A) and KW5 (B) against sodium chlorate treated (dotted bars) and non-treated (open bars) Femx and HT-29 cells. The experiment was repeated four times in duplicate. Statistics were performed by a paired t-test (GraphPad). *P *values are shown as follows: * *P *< 0.05 and *** *P *< 0.001.

In order to investigate the role of cell surface PGs for the cytotoxic effect of the peptides, the cells were incubated with sodium chlorate, an agent known to decrease the sulfation of the GAG chains [47]. The cells were incubated with sodium chlorate for 24 hours before the different peptides were added. The cytotoxic activity of the peptides against chlorate-treated and non-treated cells was investigated (Figure 2). Surprisingly, the results showed that the peptides displayed a significantly higher effect against the chlorate-treated cells compared to the non-treated cells, indicating that negatively charged GAGs at the cell surface have an inhibitory effect on the cytotoxic activity of the peptides. Incubating the cells with chlorate under the chosen conditions did not cause any reduction in cell viability, as measured by MTT assay (not shown).

### GAG deficient CHO cells were more susceptible for the peptides

**Figure 3:**
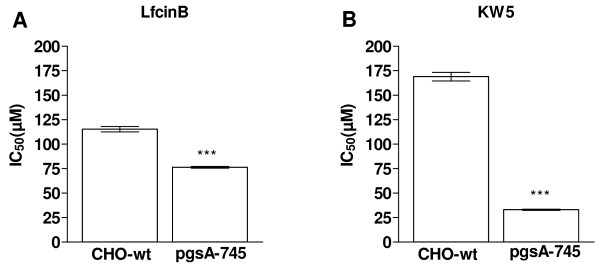
**The cytotoxic effect of LfcinB and KW5 against CHO-K1 and pgsA-745 cells**. The results are shown as the mean IC_50 _value ± SEM of LfcinB (A) and KW5 (B) against CHO-K1 and pgsA-745. The experiment was performed three times in duplicate. Statistics were performed by an unpaired t-test (GraphPad). *P *values are shown as follows: *** *P *< 0.001.

In order to verify the finding that GAGs have an inhibitory effect on the activity of the peptides, the cytotoxic effect of LfcinB and KW5 against wild type CHO cells expressing normal amounts of GAGs on the cell surface, and the complete null mutant pgsA-745 that has no expression of GAGs on the cell surface, was investigated [41]. LfcinB displayed a significantly higher activity against the GAG deficient pgsA-745 cell line compared to the wild type CHO cells, with IC_50 _values of approximately 74 μM and 112 μM, respectively (Figure 3A). KW5 also displayed a much higher activity against the GAG deficient pgsA-745 cell line compared to the wild type cells, with IC_50 _values of 32 μM and 170 μM, respectively (Figure 3B). These results confirm that GAGs expressed by the target cells inhibit the activity of the peptides.

### Determination of the type of GAGs at the cell surface

**Figure 4:**
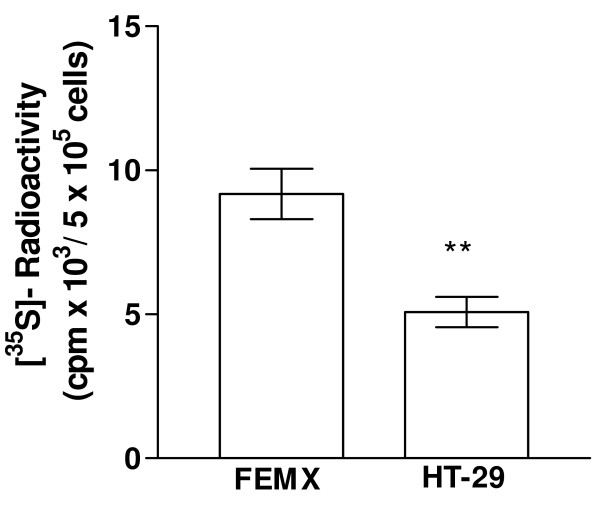
**Determination of the amount of [^35^S]sulfate incorporated into macromolecules at the cell surface of FEMX and HT-29 cells**. Data are shown as mean value ± SEM from three independent experiments performed in triplicate. Statistics were performed by an unpaired t-test (GraphPad). *P *value is shown as follows: ** *P *< 0.01.

**Figure 5:**
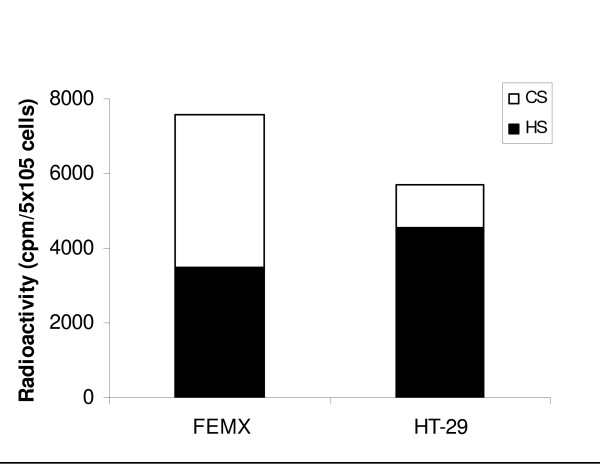
**Determination of the amount of [^35^S]sulfate incorporated into macromolecules at the cell surface of FEMX and HT-29 cells**. The experiment was performed twice in duplicate with almost identical results.

In order to compare the amount of GAGs expressed on the cell surface of FEMX and HT-29 the cells were metabolically labeled with [^35^S]sulfate. After removal of the culture medium, the GAGs associated with the plasma membrane were harvested as described in "Methods". The amount of ^35^S-labeled macromolecules in the membrane fraction was quantified after Sephadex G-50 chromatography, as previously described [48]. The ^35^S-labeled macromolecules were almost exclusively GAGs. The membrane fraction of the FEMX cells contained a higher amount (~45%) of ^35^S-labeled GAGs compared to the HT-29 cells (Figure 4). Cell surface PGs can be substituted with both CS and HS chains [49]. Previously, we have reported that LfcinB binds with higher affinity to HS than to CS [40]. The amount of HS and CS at the surface of FEMX and HT-29 cells was therefore investigated by treating the ^35^S-labeled macromolecules in the membrane fraction with cABC and HNO_2_. About 70% of the ^35^S-labeled macromolecules in the membrane fraction of the HT-29 cells were sensitive to HNO_2 _treatment, while about 10% were sensitive to cABC treatment. Hence, it can be concluded that about 70% and 10% of the ^35^S-labeled macromolecules at the surface of these cells are HS and CS, respectively. In the membrane fraction of the FEMX cells about 55% of the ^35^S-labeled material could be degraded by cABC, while about 45% could be degraded by HNO_2_-treatment (Figure 5). As can be seen from Figure 5, the HT-29 cells expressed more HS on the cell surface compared to the FEMX cells, although the FEMX cells expressed more GAGs in total (HS and CS).

### Exogenous heparin inhibited the effect of the peptides

**Figure 6:**
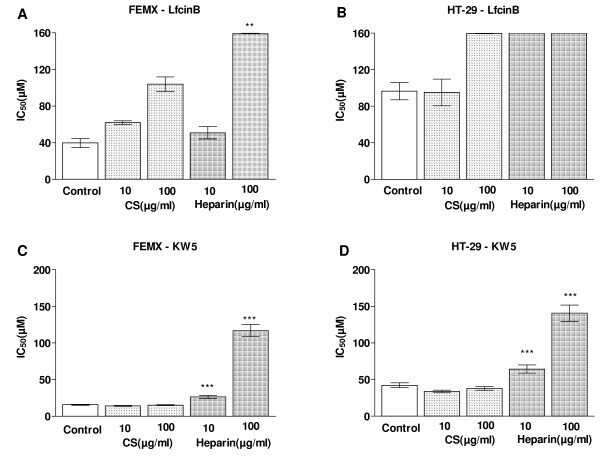
**The cytotoxic effect of LfcinB and KW5 against FEMX and HT-29 cells, in the presence of soluble heparin and chondroitin sulfate**. The results are shown as the mean IC_50 _value ± SEM (n = 5) of LfcinB and KW5 against FEMX (A) and HT-29 (B) cells, in the presence of soluble heparin and chondroitin sulfate. The IC_50 _values obtained from cell cultures containing 10 μg/ml soluble heparin were compared with cell cultures containing 10 μg/ml soluble CS by an unpaired t-test (GraphPad). The same comparison was performed between cell cultures containing 100 μg/ml soluble heparin and 100 μg/ml soluble CS. *P *values are shown as follows: ** *P *< 0.01 and *** *P *< 0.001.

The peptides displayed a lower cytotoxic activity against the HT-29 cells, which have HS as their major GAG component, compared to the FEMX cells, which have CS as their major GAG component. This, combined with the fact that the peptides bind more strongly to HS than CS [40], suggests that it is the HS component of the cell surface PGs that inhibits the cytotoxic effect of the peptides. To further investigate if the inhibitory effect was due to HS or CS, the peptides were added to cell cultures together with exogenous heparin and CS. At a concentration of 10 μg/ml, heparin and CS displayed the same inhibitory effect on the cytotoxic activity of LfcinB against the FEMX cells. However at a concentration of 100 μg/ml, heparin showed a higher inhibitory effect compared to CS (Figure 6A). The higher ability of heparin to inhibit the cytotoxic effect of LfcinB compared to CS was further demonstrated against the HT-29 cells (Figure 6B). Heparin also more efficiently inhibited the cytotoxic effect of KW5, compared to CS (Figure 6C–D). These results clearly indicate that the inhibitory effect of cell surface GAGs is due to HS, and not to CS.

### Lymphoma cells expressing HS at the cell surface were less susceptible to the cationic peptides

To further investigate the role of HS in the cytotoxic activity of the peptides, the FEMX and the HT-29 cells were treated with heparitinase, an enzyme known to depolymerize HS, before addition of the peptides. However, the results from these experiments were inconsistent, probably due to technical problems with the enzyme. An indirect approach was therefore chosen. In these experiments the cytotoxic activity of LfcinB was studied on six different lymphoma cell lines. Three of the cell lines (KMS-5, U-266, KMM-1) expressed HS at the cell surface, whereas the other three (Sudhl-4, Raji, Ramos) did not, as determined by flow cytometry using an anti-HS antibody, and as reported elsewhere (Uhlin-Hansen, L. Manuscript in preparation). The LfcinB peptide displayed a significantly higher cytotoxic activity against the HS deficient cell lines compared with the HS expressing cell lines (Table 2). The mean IC_50 _values obtained against the HS deficient cell lines and the HS expressing cell line was 13 μM ± 2 and 50 μM ± 6, respectively (*P *value 0.004) This experiment further supports the finding that HS at the cell surface inhibits the cytotoxic activity of the peptides.

**Table 2:** The cytotoxic effect of LfcinB against lymphoma cell lines expressing different levels of HS.

Cell line	^a^Cell surface HS	^b^IC_50_(μM)
KMS-5	+	38
U-266	+	55
KMM-1	+	57
Sudhl-4	-	16
Raji	-	13
Ramos	-	10

^a^Cell surface HS was measured using an anti-HS antibody and flow cytometry. Cell lines marked with + express HS, while cell lines marked with -lack HS.

^b^IC_50 _is the inhibitory concentration of the LfcinB peptide in (μM) where 50% of the cells are killed.

### HT-29 cells synthesize larger PGs than FEMX cells

**Figure 7:**
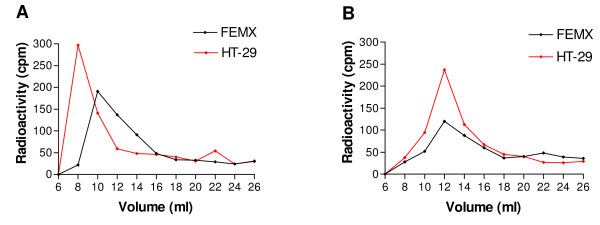
**Superose 6 gel chromatography of ^35^S-labeled macromolecules from the plasma membrane fraction before and after alkali treatment**. **A) **Intact ^35^S-labeled PGs and **B) **^35^S-labeled GAGs, obtained by alkali treatment. The experiment was performed twice with similar results.

In addition to the type of GAGs, other features such as the size of the GAG chains might affect the cytotoxic effect of the peptides. The size of the proteoglycan molecules and their GAG chains was therefore analyzed by Superose-6 gel chromatography. The ^35^S-labeled macromolecules from the membrane fraction of the FEMX cells and the HT-29 cells eluted with peak k_av _values of 0, 33 and 0, 25, respectively (Figure 7A). This shows that the HT-29 cells expressed larger proteoglycan molecules at the cell surface, compared to the FEMX cells. Chromatography of corresponding ^35^S-labeled material after alkali treatment revealed a shift in the elution of the peak fractions (Figure 7B), confirming that the GAGs in the membrane fractions were part of PG molecules. The size of the attached ^35^S-labeled GAG chains from the HT-29 and FEMX cells was almost identical, eluting with peak k_av_-values of 0,38 and 0,40 respectively. These k_av_-values correspond to molecular weight of approximately 48 kDa and 45 kDa. These results indicate that the larger size of the PGs in HT-29 cells, compared to the FEMX cells, is due to more GAG chains attached to each core protein, or larger core proteins.

## Discussion

It is known that a variety of lytic peptides show a selective cytotoxic activity against tumor cells compared to normal cells. It is believed that this selectivity is due to a more negatively charged cell surface of the tumor cells, but the exact mechanism is not known. The plasma membrane of almost all mammalian cells contains PGs substituted with GAG chains. The GAGs are highly anionic due to a large number of negatively charged sulfate groups [28]. We have previously shown that LfcinB and other lytic peptides bind to GAGs [27] and we therefore hypothesized that cell surface GAGs increase binding and therefore in accordance with work on other anionic membrane components increase the cytotoxic activity of CAPs. However, the present study showed that cell surface GAGs inhibited the cytotoxic effect of LfcinB and the designer lytic peptide, KW5.

In the initial experiments we used two cell lines which express different amounts of GAGs at the cell surface; the melanoma cell line FEMX and the colon carcinoma cell line HT-29. Both peptides displayed higher cytotoxic activity against the FEMX cells, which express a larger amount of GAGs compared to the HT-29 cells. This result is in agreement with previous studies showing that an increased negative charge on the cell surface of target cells enhances the cytotoxic activity of lytic peptides [50,51]. To confirm our hypothesis that it is the negatively charged sulfate groups on the GAG chains that mediate the binding of the peptides, the sulfation of the GAGs was reduced by adding sodium chlorate to the culture medium before the cells were exposed to the peptides. Chlorate reduces the overall sulfation of GAGs by competing with sulfate ions for binding to ATP-sulfyrolase [52]. Interestingly, we found that the peptides displayed a significantly greater cytotoxic activity against chlorate-treated cells compared to non-treated cells, indicating that the negatively charged GAGs at the cell surface actually reduce the cytotoxic activity of the peptides. Previous studies have shown that incubating cells with sodium chlorate only leads to a partial reduction of the sulfation of the GAG chains [53]. This may explain why the effect of chlorate on the cytotoxic effect was only moderate.

In order to investigate the involvement of GAGs more directly, the cytotoxic activity of the peptides was studied with wild type and GAG-defective CHO cells [41]. These cells have been widely used to study the role of cell surface GAGs in various processes such as viral infection, growth factor signaling and cell adhesion [54]. The pgsA-745 cells have defective xylosyltransferase, an enzyme necessary for biosynthesis of HS and CS [41]. The higher cytotoxic activity of the peptides against the GAG deficient pgsA-745 cells compared to the wild type CHO cells expressing normal levels of GAGs at the cell surface clearly indicate that GAGs have an inhibitory effect on the cytotoxic activity of these peptides. This finding is in contrast to reports about other anionic cell surface molecules which have been shown to enhance the anticancer activity of CAPs [50,51].

Cell surface PGs may be substituted with different types of GAG chains, either HS or CS [28]. We have previously shown that LfcinB and other lytic peptides bind with higher affinity to HS than CS [40]. We therefore examined the expression pattern of GAGs on the FEMX and HT-29 cells. We found that the FEMX cells expressed cell surface PGs mostly substituted with CS, whereas the PGs at the surface of HT-29 cells were mostly substituted with HS. This expression profile is consistent with previous reports showing that malignant melanoma cells have a high expression of CSPGs [55], while the HT-29 cells has a high expression of HSPGs. [56]. The lower cytotoxic activity of the peptides against the HT-29 cells compared to the FEMX cells therefore suggests that the inhibitory effect of GAGs is attributed to HS and not CS. The addition of soluble heparin and CS to cell cultures is widely used to study the interaction between various molecules and cell surface HS and CS [57,58]. In the present study we found that exogenously added heparin had a stronger inhibitory effect on the cytotoxic activity of the peptides, compared to CS. This experiment strongly supports the finding that it is HS, and not CS, that inhibits the cytotoxic activity of the peptides. The inhibitory effect of HS was further supported by the fact that lymphoma cells lacking HS at the cell surface were much more sensitive to LfcinB than lymphoma cells expressing cell surface HS.

**Figure 8:**
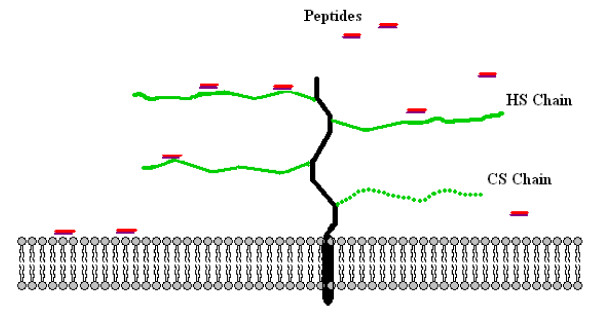
**Schematic model of the interaction of HS chains with CAPs on the cell surface**.

Cell surface PGs belong to two different families, the syndecans and the glypicans [59,60]. They can be substituted with both HS and CS side chains, but HS is the dominant type of GAG on the surface of most cell types [61]. The complex polysaccharide structure gives HS a higher conformational flexibility compared to CS [62]. This may explain the fact that LfcinB and other lytic peptides bind with higher affinity to HS than to CS since the sequence and structural diversity in the lytic peptides may require a high flexibility in the molecules they bind to. Previous studies have shown that the antibacterial activity of the peptides LL-37 [63,64] and α-defensin [65] are inactivated by GAGs. It has also been demonstrated that the LfcinB peptide and a set of short α-helical peptides are able to block HSV infection by binding to cell surface HS, which is used as a target molecule for HSV internalization [40,66,67]. It is believed that PGs with their unbranched GAG chains extend like a brush out from the surface of the cells, as shown in the cartoon in Figure 8. As discussed, our results show that HS at the surface of the target cells inhibit the cytotoxic activity of the peptides. We therefore propose that the long, unbranched HS chains can sequester the lytic peptides away from the phospholipid bilayer and thereby impede their ability to induce cytolysis of the target cells, as shown in Figure 8.

An earlier finding showing that low-molecular heparin is less efficient in binding lactoferrin than normal sized heparin [68], indicates that the size of the GAG chains could be an important factor for the interaction with the peptides. In the present study we found the size of the PGs on the surface of the HT-29 cells to be larger than the PGs on the surface of the FEMX cells. Larger PGs may keep the lytic peptides at a longer distance from the phospholipid bilayer than smaller PGs. The size of the cell surface PGs may therefore influence the sensitivity of the cells for lytic peptides. In addition to the size of the proteoglycans the degree and pattern of sulfation of the GAG chains may also influence the level of interaction between the peptides and the GAG chains. If the peptides have high sequence specificity and requires a particular sulfation pattern in order to interact with the GAGs this would also affect their binding capacity as shown for growth factors [69,70], antithrombin III [71] and matrix ligand [72].

It has been shown that phosphatidylserine [51] and sialic acid [50] found on the cell surface of tumor cells enhance the antitumor activity of CAPs. Phosphatidylserine is present at the outer leaflet of many cancer cell lines and a higher number of sialic acids is present on glycoproteins and glycolipids on cancer cells compared to normal cells. Thus, these smaller anionic molecules located closer to the phospholipid bilayer of the tumor cells might facilitate the activity of the peptides whereas the large PGs have an opposite effect.

Many studies have shown that poorly differentiated tumors have reduced expression levels of cell surface HSPGs [35], indicating that the biosynthesis of HS PGs is reduced in these tumors. The best studied cell surface proteoglycan is syndecan-1. In a wide range of carcinomas it has been shown that a low expression of syndecan-1 on tumor cell surfaces correlates with increasing metastatic potential and poor prognosis [73-75]. In addition to reduced synthesis, an increased degradation of cell surface HS may take place. Heparanase is a HS degrading enzyme released by several cell types [76]. It has been found that many tumors overexpress heparanase [77]. Further, increased expression of heparanase is correlated with the high metastatic capacity of tumor cells [77]. Heparanase released by the tumor cells degrades HS both in the extracellular matrix and at the cell surface. Hence, the low level of cell surface HS found in many tumors can be a result of increased level of heparanase. Alterations in the expression pattern of HS at the surface of tumor cells, due to reduced biosynthesis of HS and/or increased expression of heparanase, can explain, at least partly, the fact that many cancer cells show increased susceptibility toward certain CAPs.

## Conclusion

In the present study we show for the first time that negatively charged molecules on the surface of tumor cells can inhibit the antitumor activity of CAPs. Further studies should be done with other structurally different CAPs to conclude whether this is the case for all types of CAPs. We show that the cytotoxic activity was significantly reduced in cells expressing HS at the surface. Our results indicate that HS at the surface of cancer cells can sequester the CAPs away from the phospholipid bilayer and thereby hinder their ability to induce cytolysis. Previous studies have shown that low level of cell surface HS is correlated with high metastatic potential of cancer cells. Our results indicate that poorly differentiated tumors, with low expression of cell surface HS, are more susceptible to treatment with CAPs. To confirm this, the effect of CAPs on tumors with high and low HS expression should be explored with *in vivo *studies.

## Competing interests

The authors declare that they have no competing interests.

## Authors' contributions

BF carried out all experiments, including cell culture, cytotoxicity assays, and chromatography. ØR and LUH designed the study. All authors contributed in the discussion and interpretation of the results and writing the manuscript.

## Pre-publication history

The pre-publication history for this paper can be accessed here:

http://www.biomedcentral.com/1471-2407/9/183/prepub
